# Marine Ranching Systems Exhibit Higher Multi-Trophic Biodiversity: Evidence from Three Ranching Areas

**DOI:** 10.3390/ijms27031483

**Published:** 2026-02-02

**Authors:** Kui Zhang, Xue Sun, Hui Jia, Cui Liang, Hui Zhang

**Affiliations:** 1 College of Life Sciences, Qingdao Agricultural University, Qingdao 266109, China; zhangkui@qdio.ac.cn (K.Z.); sunxue@qdio.ac.cn (X.S.); 2 Laboratory of Marine Ecology and Environmental Sciences, Institute of Oceanology, Chinese Academy of Sciences, Qingdao 266071, China; jiahui@qdio.ac.cn; 3 School of Marine Sciences, Ningbo University, Ningbo 315823, China; 4 University of Chinese Academy of Sciences, Beijing 100049, China

**Keywords:** marine ranching, eDNA metabarcoding, environmental factors, biodiversity monitoring

## Abstract

Marine ranching has become an important strategy for offshore ecological restoration and fisheries resource conservation in China. In this study, environmental DNA (eDNA) was applied to simultaneously monitor phytoplankton, invertebrates, and fish communities in the Tianjin Dashentang Marine Ranching, the Tianjin Binhai National Marine Park, and the Western Furong Island Marine Ranching Area. eDNA analyses detected more than 190 phytoplankton species, over 340 invertebrate species, and approximately 100 fish species across the three regions. Species richness and community diversity were consistently higher within marine ranching zones than in adjacent control areas, and ranching zones supported a higher proportion of endemic and ecologically important taxa. Redundancy analysis identified temperature, salinity, and pH as the main environmental drivers shaping community composition. Temperature had stronger effects on phytoplankton and invertebrate assemblages in the Dashentang and Furong Island ranches, whereas pH and conductivity were more influential in the Binhai National Marine Park. Temporal comparisons of fish eDNA data from 2021 to 2024 indicated increased alpha diversity, greater representation of key taxa, and higher community stability in 2024. Overall, these results demonstrate the utility of eDNA for integrated biodiversity monitoring and provide scientific support for evaluating and guiding marine ranching development in the Bohai Sea region.

## 1. Introduction

In recent years, coastal fishery resources in China have significantly declined due to overfishing, environmental pollution, and habitat destruction [1]. Traditional fishery models struggle to balance resource conservation with economic development needs, necessitating a shift toward ecological and sustainable approaches [2]. As a new approach integrating ecological restoration with fishery enhancement [3], marine ranching has been rapidly promoted nationwide. Marine ranching refers to optimizing marine ecosystems through ecological restoration, artificial stock enhancement, and fishery infrastructure development in designated sea areas. This creates suitable environments for marine organisms’ growth, reproduction, and habitat, ultimately achieving both fishery resource enhancement and increased income for fishermen [4]. The development of marine ranching in China has progressed rapidly, starting from experimental small-scale fish reefs in 1979 to the successive approval of 153 national-level marine ranch demonstration areas across the Bohai Sea, Yellow Sea, East China Sea, and South China Sea by 2021 [1].

The Bohai Sea, a typical semi-enclosed marine area, is characterized by intense human activity and a fragile ecosystem [5]. It is also one of China’s key regions for marine ranch construction and ecological restoration [3]. In recent years, national demonstration projects for marine ranches and inshore ecological restoration have supported construction efforts along the Bohai coast that center on deploying artificial reefs [6], restoring shellfish reefs [7], and rehabilitating habitats [8]. These efforts have improved local habitat conditions and promoted the recovery of fishery resources.

As marine ranching expands, rigorous evaluation of its ecological restoration outcomes and potential risks has become increasingly important. Previous work has shown that marine ranches can increase fish biomass and population density to some extent [9]. However, ecological effects are not confined to a single taxonomic group [10]. Continuous, real-time monitoring of phytoplankton, invertebrate, and fish communities is still required to prevent problems such as invasive species [11] or nutritional imbalances [12]. At present, the effectiveness of marine ranch construction relies on precise, quantitative monitoring [13].

Conventional in situ surveys are often labor-intensive, time-consuming, and costly, and may be constrained by meteorological and logistical conditions [14]. However, traditional in situ approaches remain valuable for documenting species richness and detecting cryptic taxa that might be overlooked by simpler sampling methods [15]. Environmental DNA (eDNA) metabarcoding has increasingly been shown to provide an efficient, non-invasive, and complementary tool to conventional surveys, helping to capture additional biodiversity signals that may be missed by visual or gear-based methods alone [14].

In 2024, this study was conducted to investigate the community structures of fish, invertebrates, and phytoplankton in the Tianjin Dashentang Marine Ranching, the oyster reef area of the Tianjin Binhai National Marine Park, and the Western Furong Island Marine Ranching Area using multi-primer eDNA metabarcoding. The specific objectives were to: (1) characterize multitrophic biodiversity across the three marine ranches, providing baseline data to support the further development of marine ranching; and (2) identify key environmental variables shaping biological community distribution patterns through integrated environmental factor analysis.

## 2. Results

### 2.1. Overview of eDNA Sequencing Data

A total of 28 water samples were collected from three marine ranching areas during May, June, and August 2024. The three metabarcoding assays collectively produced 6,528,555 sequencing reads: 18S V4 yielded 2,428,601 reads, 12S yielded 1,917,307 reads, and 18S V9 yielded 2,182,647 reads. No positive records were detected in the negative controls. After quality control, high-quality reads numbered 2,020,703 for 18S V4, 1,745,256 for 12S, and 1,985,251 for 18S V9. In the waters of the Tianjin Dashentang Marine Ranching, the 18S V4, 12S, and 18S V9 metabarcoding reads were classified into 1496 ASVs, 1143 ASVs, and 643 OTUs, respectively. Monitoring detected 126 phytoplankton species across 62 families and 71 genera, 76 fish species across 59 families and 93 genera, and 219 invertebrate species across 147 families and 173 genera. In the waters of oyster reef area of the Tianjin Binhai National Marine Park, the three assays produced 1769 ASVs, 702 ASVs, and 182 OTUs, respectively. Monitoring detected 149 phytoplankton species, 43 fish species, and 160 invertebrate species. In the waters of the Western Furong Island Marine Ranching Area, the three assays produced 1660 ASVs, 1183 ASVs, and 213 OTUs, respectively. Monitoring detected 189 phytoplankton species, 52 fish species, and 229 invertebrate species.

### 2.2. Species Composition Across Marine Ranches

#### 2.2.1. Tianjin Dashentang Marine Ranching

**Figure 1:**
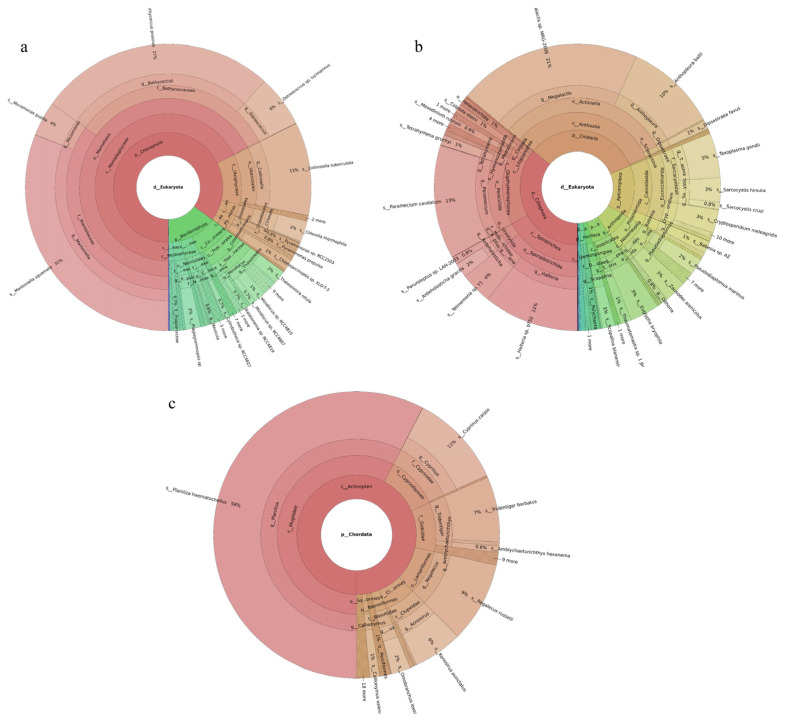
Species composition and relative abundance at different taxonomic levels in the Tianjin Dashentang Marine Ranching. (**a**): phytoplankton; (**b**): invertebrates; (**c**): fish. Different colors represent different taxonomic groups. Taxa with very low relative abundance are not individually shown in the figure. Detailed taxonomic information is provided in Supplementary Materials Table S1.

Three phytoplankton phyla were revealed. Chlorophyta was the most abundant phylum, accounting for 85% of the total, followed by Bacillariophyta. Of the 126 phytoplankton species identified, the dominant species were *Mantoniella squamata* (31%), *Bathycoccus prasinos* (27%), *Collinsiella tuberculata* (11%), *Ostreococcus* sp. *Lucimarinus* (6%), and *Micromonas pusilla* (4%) (Figure 1a). Seventy-six fish species were identified. The dominant species were *Planiliza haematocheilus* (58%), *Cyprinus carpio* (11%), *Regalecus russelii* (9%), *Tridentiger barbatus* (7%), and *Konosirus punctatus* (6%) (Figure 1c). Nineteen invertebrate phyla were identified. Ciliophora was the most abundant phylum, accounting for 36%, followed by Cnidaria at 33%. Of the 219 invertebrate species recorded, the dominant taxa were *Megalactis* sp. *MRG-2006* (21%), *Paramecium caudatum* (13%), *Halteria* sp. *bTS2* (11%), *Anthopleura ballii* (10%), and *Toxoplasma gondii* (5%) (Figure 1b).

#### 2.2.2. Tianjin Binhai National Marine Park

**Figure 2:**
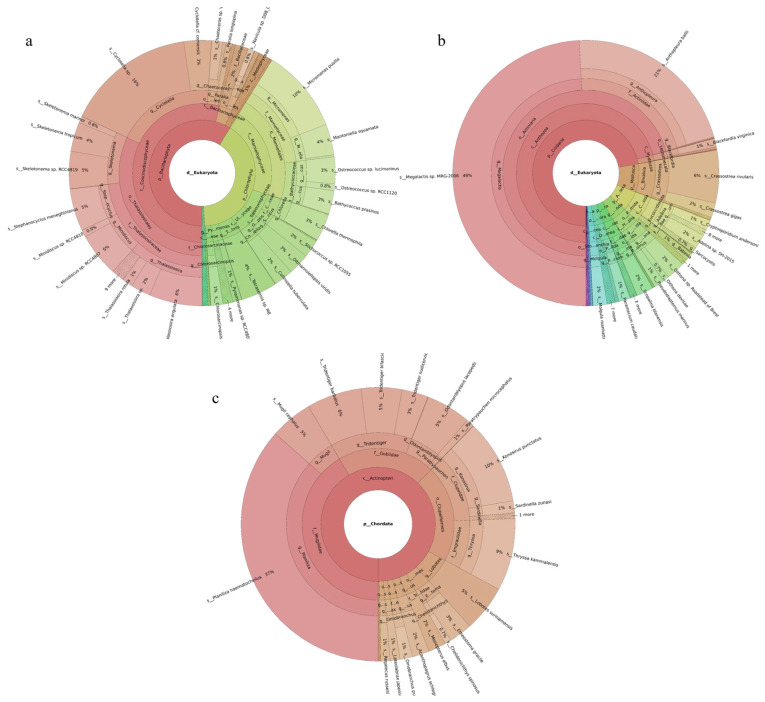
Species composition and relative abundance at different taxonomic levels in the Tianjin Binhai National Marine Park. (**a**): phytoplankton; (**b**): invertebrates; (**c**): fish. Different colors represent different taxonomic groups. Taxa with very low relative abundance are not individually shown in the figure. Detailed taxonomic information is provided in Supplementary Materials Table S2.

Phytoplankton belonging to five phyla were revealed, with Bacillariophyta accounting for the largest share (59%) and Chlorophyta the next largest (40%). Among the 149 revealed phytoplankton species, *Cyclotella* sp. (16%), *Micromonas pusilla* (10%), *Thalassiosira angulata* (6%), and *Stephanocyclus meneghinianus* (5%) were dominant (Figure 2a). Forty-three fish species were detected, with *Planiliza haematocheilus* (37%), *Konosirus punctatus* (10%), *Thryssa kammalensis* (9%), and *Tridentiger barbatus* (6%) identified as dominant (Figure 2c). The 160 detected invertebrate species belonged to 19 phyla; Cnidaria predominated (72%), followed by Mollusca (9%). The dominant invertebrate species were *Megalactis* sp. *MRG-2006* (49%), *Anthopleura ballii* (22%), and *Crassostrea rivularis* (6%) (Figure 2b).

#### 2.2.3. Western Furong Island Marine Ranching Area

**Figure 3:**
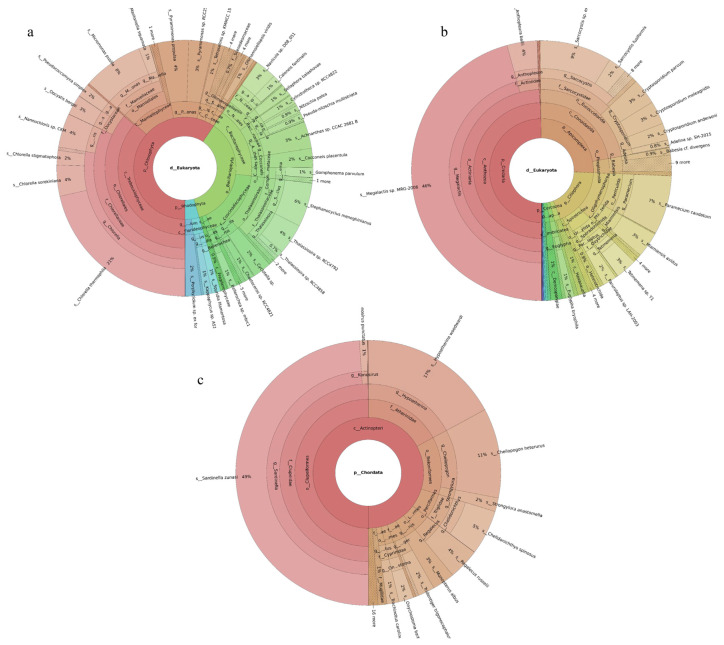
Species composition and relative abundance at different taxonomic levels in the Western Furong Island Marine Ranching Area. (**a**): phytoplankton; (**b**): invertebrates; (**c**): fish. Different colors represent different taxonomic groups. Taxa with very low relative abundance are not individually shown in the figure. Detailed taxonomic information is provided in Supplementary Materials Table S3.

A total of 189 phytoplankton species were assigned to four phyla. Chlorophyta accounted for the largest share (60%), followed by Bacillariophyta (36%). The most abundant species were *Chlorella thermophila* (21%), *Micromonas pusilla* (8%), and *Stephanocyclus meneghinianus* (6%) (Figure 3a). 52 fish species were detected, with *Sardinella zunasi* (49%) showing the highest relative abundance, followed by *Hypoatherina woodwardi* (17%), *Cheilopogon heterurus* (11%), and *Chelidonichthys spinosus* (5%) (Figure 3c). Among the 229 invertebrate species recorded, representatives spanned 44 phyla. Cnidaria constituted the largest proportion (50%), followed by Apicomplexa (26%). The dominant taxa were *Megalactis* sp. *MRG-2006* (46%), *Sarcocystis* sp. *ex Pantherophis alleghaniensis* (9%), and *Paramecium caudatum* (7%) (Figure 3b).

### 2.3. Differences in Community Composition Among Marine Ranches

**Figure 4:**
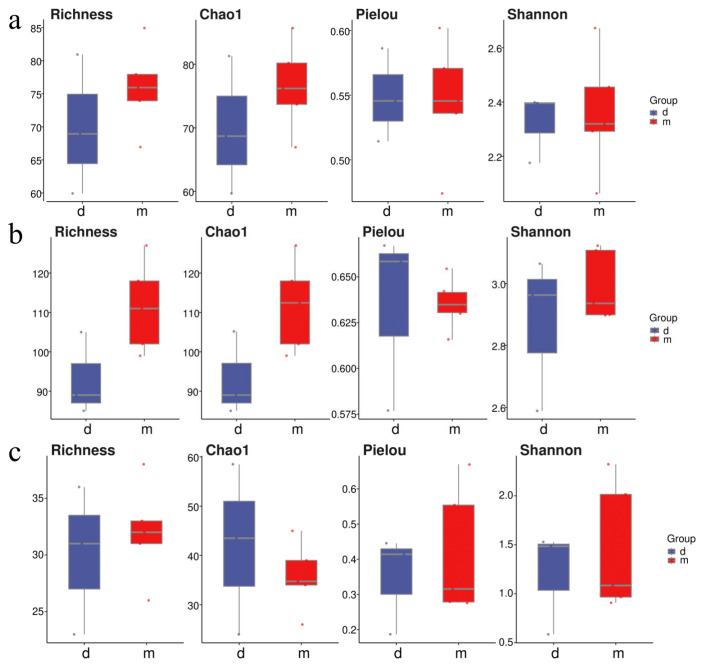
Alpha diversity indices in the Tianjin Dashentang Marine Ranching. (**a**): Phytoplankton; (**b**): invertebrates; (**c**): fish. “d” represents Control area; “m” represents Ranching zone. The dots represent the values of the alpha diversity indices for individual samples.

**Figure 5:**
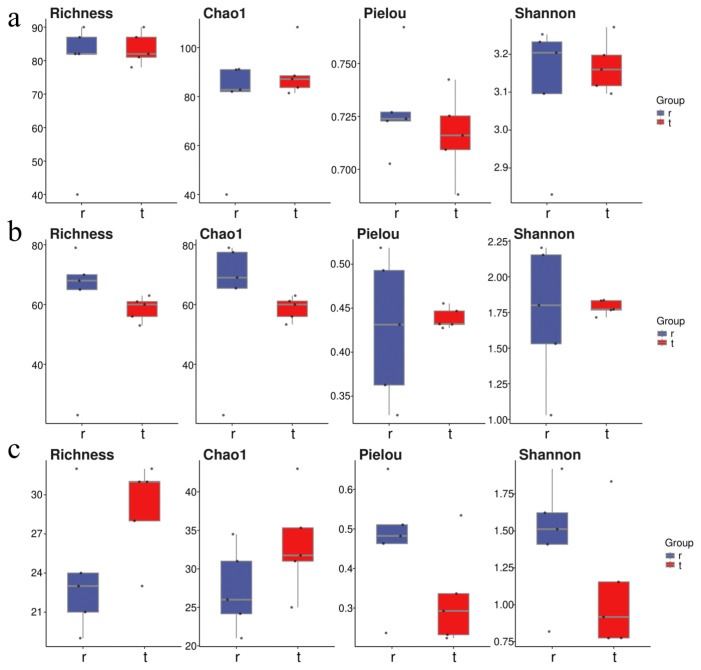
Alpha diversity indices in the Tianjin Binhai National Marine Park. (**a**): Phytoplankton; (**b**): invertebrates; (**c**): fish. “r” represents Artificial Oyster Reef Area; “t” represents Natural Oyster Reef Area. The dots represent the values of the alpha diversity indices for individual samples.

**Figure 6:**
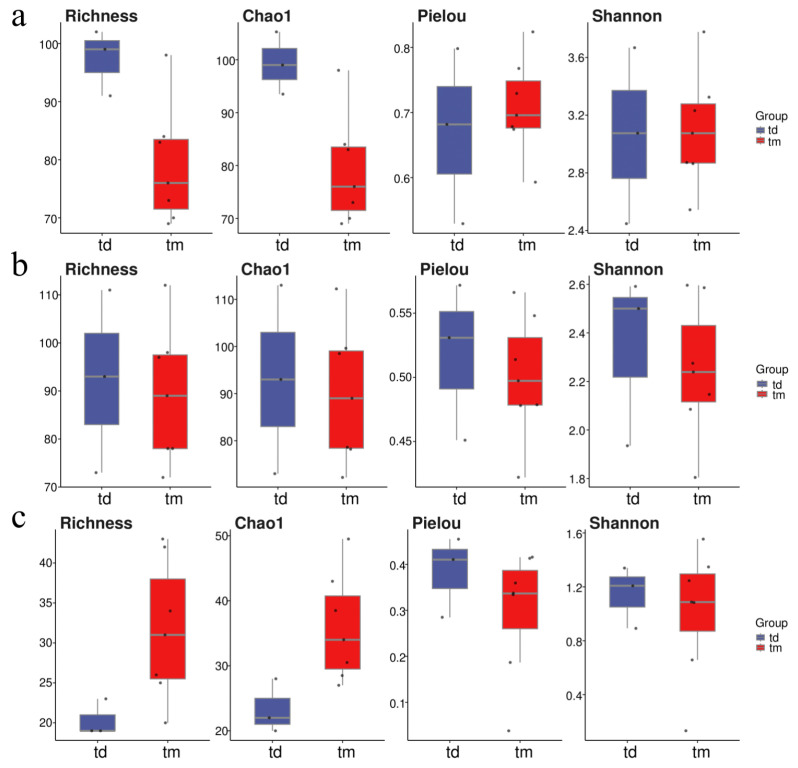
Alpha diversity indices in the Western Furong Island Marine Ranching Area. (**a**): Phytoplankton; (**b**): invertebrates; (**c**): fish. “tm” represents Ranching zone; “td” represents Control area. The dots represent the values of the alpha diversity indices for individual samples.

At the Tianjin Dashentang Marine Ranching area (Figure 4), Richness and Chao1 indices indicated higher species richness for phytoplankton and invertebrates in the ranching zone than in the control area, but these differences were not statistically significant (Richness: *p* > 0.05; Chao1: *p* > 0.05). Similarly, Shannon and Pielou indices showed no significant differences in evenness for any of the three major taxonomic groups between the ranching and control zones (all *p* > 0.05). At the Tianjin Binhai National Marine Park (Figure 5), fish and phytoplankton exhibited higher Richness and Chao1 values in the natural reef area compared with the artificial reef area, whereas invertebrates had higher Richness and Chao1 in the artificial reef. Although Shannon and Pielou indices for fish were numerically greater in the artificial reef than in the natural reef, none of the four α diversity indices across the three major taxa differed significantly between the two reef types (all *p* > 0.05). In contrast, at the Western Furong Island Marine Ranching Area (Figure 6), richness and Chao1 indices for fish and invertebrates were significantly higher in the ranching zone than in the control area (*n* = 10, Richness: *p* < 0.05; Chao1: *p* < 0.05), whereas for phytoplankton both Richness and Chao1 values were significantly higher in the control area (*n* = 10, *p* < 0.05). Although Shannon and Pielou indices were numerically higher in the control area for all three taxonomic groups, these differences did not reach statistical significance (all *p* > 0.05).

**Figure 7:**
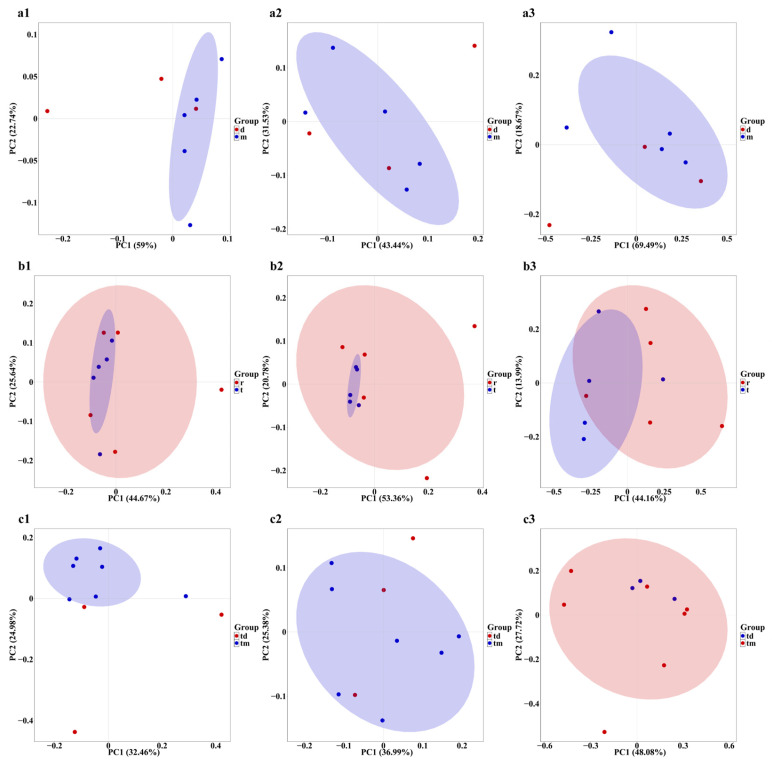
PCoA of community composition across the three study areas. (**a1**–**a3**): Tianjin Dashentang Marine Ranching: (**a1**) phytoplankton, (**a2**) invertebrates, (**a3**) fish; (**b1**–**b3**) Tianjin Binhai National Marine Park: (**b1**) phytoplankton, (**b2**) invertebrates, (**b3**) fish; (**c1**–**c3**) Western Furong Island Marine Ranching Area: (**c1**) phytoplankton, (**c2**) invertebrates, (**c3**) fish. “d” represents the control area; “m” represents the ranching zone; “r” represents the artificial oyster reef area; “t” represents the natural oyster reef area; “tm” represents the ranching zone; “td” represents the control area. The colored areas represent 95% confidence ellipses for different groups.

Principal Coordinates Analysis (PCoA) based on Bray–Curtis distance indicated that sampling points in the Dashentang Marine Ranching zone were more tightly clustered, whereas those in the control area were more dispersed, although overall community differences were minimal (Figure 7(a1–a3)). In the oyster reef area of the Tianjin Binhai National Marine Park, sampling points from the natural reef showed stronger aggregation across all three biological groups, while communities associated with the artificial reef were more dispersed (Figure 7(b1–b3)). At the Furong Island Marine Ranching zone, phytoplankton sampling points were more concentrated, whereas fish sampling points were more clustered in the control area (Figure 7(c1–c3)).

**Figure 8:**
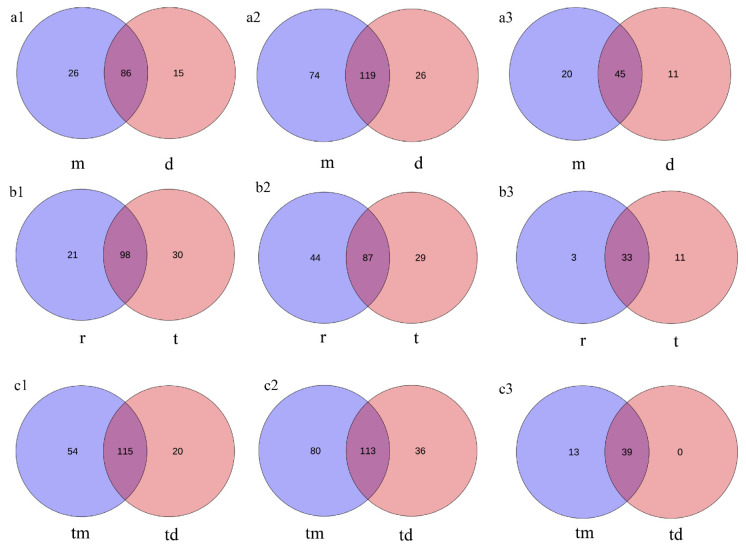
Venn diagram of species overlap across the three study areas. (**a1**–**a3**) Tianjin Dashentang Marine Ranching: (**a1**) phytoplankton, (**a2**) invertebrates, (**a3**) fish; (**b1**–**b3**) Tianjin Binhai National Marine Park: (**b1**) phytoplankton, (**b2**) invertebrates, (**b3**) fish; (**c1**–**c3**) Western Furong Island Marine Ranching Area: (**c1**) phytoplankton, (**c2**) invertebrates, (**c3**) fish. “d” represents the control area; “m” represents the ranching zone; “r” represents the artificial oyster reef area; “t” represents the natural oyster reef area; “tm” represents the ranching zone; “td” represents the control area.

Venn diagrams show that within the Tianjin Dashentang Marine Ranching, the ranching zone contains approximately 1.73, 2.85, and 1.82 times more unique species of phytoplankton, invertebrates, and fish, respectively, than the control area (Figure 8(a1–a3)). In the Tianjin Binhai National Marine Park, the natural reef area predominates in unique phytoplankton and fish species, while the artificial reef area contains more unique invertebrate species (Figure 8(b1–b3)). At the Western Furong Island Marine Ranching Area, the ranching zone contains 2.7 times more unique phytoplankton species and 2.22 times more unique invertebrate species than the control area. Moreover, exclusive fish species were detected only within the ranching zone; no unique fish species were observed in the control area (Figure 8(c1–c3)).

### 2.4. Environmental Drivers of Community Composition

**Figure 9:**
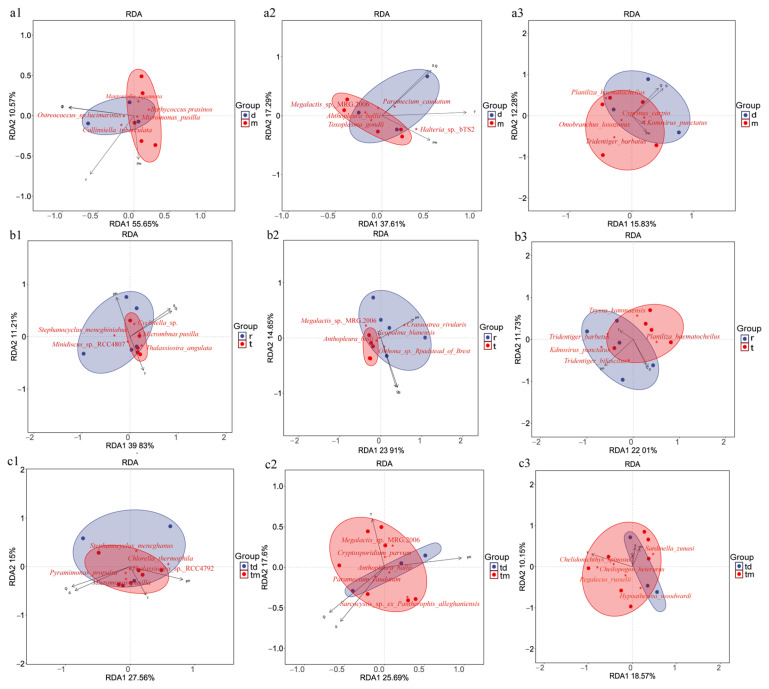
RDA of community composition across the three study areas. (**a1**–**a3**) Tianjin Dashentang Marine Ranching: (**a1**) phytoplankton, (**a2**) invertebrates, (**a3**) fish; (**b1**–**b3**) Tianjin Binhai National Marine Park: (**b1**) phytoplankton, (**b2**) invertebrates, (**b3**) fish; (**c1**–**c3**) Western Furong Island Marine Ranching Area: (**c1**) phytoplankton, (**c2**) invertebrates, (**c3**) fish. “d” represents the control area; “m” represents the ranching zone; “r” represents the artificial oyster reef area; “t” represents the natural oyster reef area; “tm” represents the ranching zone; “td” represents the control area. Arrows indicate environmental vectors, including salinity (S), conductivity (Q), temperature (T), and pH; the direction and length of each arrow reflect the strength and direction of correlations with community composition. Red five-pointed stars represent key species showing the strongest responses to environmental gradients in the RDA analysis. Colored areas denote the 95% confidence ellipses for different groups.

Redundancy analysis (RDA) was used to examine the relationships between environmental variables and community composition across the three study areas. In the Tianjin Dashentang Marine Ranching (Figure 9a), the first two RDA axes (RDA1 and RDA2) explained 66.27% of phytoplankton community variation, with temperature as the dominant factor; samples from the ranching zone were more sensitive to pH. Invertebrate community variation was explained at 54.9%, with temperature as the dominant driver. Fish community variation was explained at 28.11%, with salinity as the primary influence. In the oyster reef area of the Tianjin Binhai National Marine Park (Figure 9b), phytoplankton community variation accounted for 51.04% of the total variation, with pH as the dominant factor. Invertebrate community variation explained 38.56%, with salinity and conductivity exerting comparable influence. Fish community variation accounted for 33.74%, with pH as the dominant factor. In the Western Furong Island Marine Ranching Area (Figure 9c), phytoplankton community variation explained 42.56% of the total variation and was primarily governed by salinity and conductivity; samples from the ranching zone showed increased sensitivity to temperature. Invertebrate community variation accounted for 43.2%, with temperature as the dominant factor. Fish community variation explained 28.72%, with temperature as the principal governing factor.

### 2.5. Temporal Changes in Fish Community Structure

**Figure 10:**
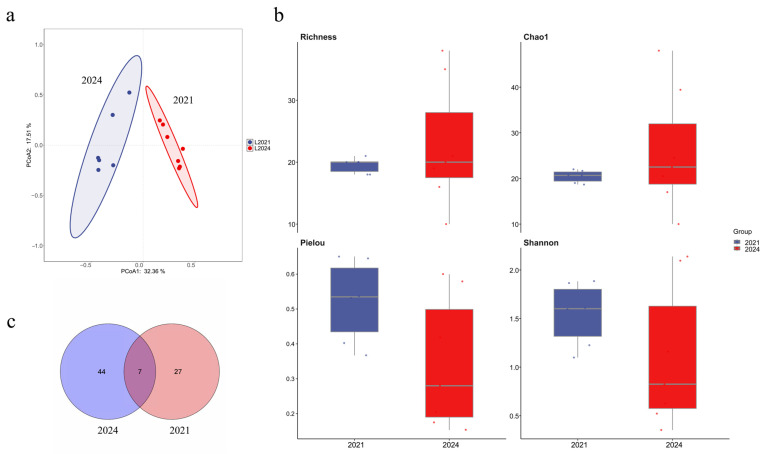
Comparison of fish community structure between 2021 and 2024 in the Western Furong Island Marine Ranching Area. (**a**): PCoA of community composition; The colored areas represent the 95% confidence ellipses for different groups. (**b**): Alpha diversity indices; The dots represent the values of the alpha diversity indices for individual samples. (**c**): Venn diagram showing shared and unique species.

To assess long-term effects of marine ranching development in the Western Furong Island Marine Ranching Area, this study compared 2024 fish eDNA monitoring data with historical data from 2021. The number of endemic fish species in 2024 significantly increased relative to 2021. Dominant species in 2024 were *Hypoatherina woodwardi*, *Cheilopogon heterurus*, *Regalecus russelii*, *Strongylura anastomella*, *Trachinotus carolinus*, and *Sardinella zunasi*; in 2021 the dominant species were *Sardinella zunasi*, *Planiliza haematocheilus*, and *Thryssa kammalensis*. The Venn diagram shows a larger set of unique species in 2024 than in 2021 (Figure 10c). Alpha diversity analysis revealed higher species richness in 2024 than in 2021 (Figure 10b). PCoA demonstrated significant differences between the fish communities of the two years, with 2024 samples showing a more concentrated distribution (Figure 10a).

## 3. Discussion

A comprehensive understanding of biodiversity changes in marine ranching systems is essential for effective management and sustainable development, as marine ranching represents an important approach for marine ecological restoration and fisheries resource conservation [3]. Previous studies have largely focused on single taxonomic groups within individual marine ranches, such as invertebrates [16] or fish [17], thereby limiting insights into multitrophic biodiversity patterns. In this study, environmental DNA (eDNA) technology was applied to monitor biodiversity in ranching zones and adjacent control areas of the Tianjin Dashentang Marine Ranching and the Western Furong Island Marine Ranching Area, as well as in artificial-reef and natural-reef areas within the Tianjin Binhai National Marine Park. A comparative analysis was conducted to examine biodiversity and community structure across three major taxonomic groups—phytoplankton, invertebrates, and fish. Overall, marine ranching zones exhibited higher biodiversity and more complex community composition, consistent with the role of artificial reefs in enhancing habitat complexity and the positive effects of marine ranching management on species recovery [18].

### 3.1. Multitrophic Biodiversity Revealed by eDNA Metabarcoding

Multi-marker eDNA metabarcoding has been widely applied in marine biodiversity monitoring because targeting multiple gene fragments improves taxonomic coverage and resolution across phytoplankton, invertebrate, and fish communities [19]. Compared with single-marker approaches, this strategy enhances the monitoring of rare or cryptic species [20] and reduces marker-specific biases [21], thereby increasing the reliability of biodiversity assessments. In marine ecosystems, multi-marker eDNA approaches have been successfully applied in diverse ecological contexts, including seamounts [22] and cold seep systems [23]. Primer performance varies among taxonomic groups, with broad-spectrum 18S rRNA primers commonly used for general eukaryotic surveys such as phytoplankton and invertebrates [24], whereas mitochondrial 12S rRNA primers exhibit high specificity for fish and have become a standard tool for fish eDNA monitoring [25]. By integrating multiple genetic markers, multi-marker eDNA metabarcoding achieves a balance between taxonomic breadth and resolution, enabling comprehensive biodiversity monitoring in complex marine ecosystems and supporting applications such as invasive species monitoring [26] and climate change impact assessment [27]. Nevertheless, the interpretation of multi-marker eDNA metabarcoding results requires consideration of several taxon-specific methodological limitations. Detection probability and taxonomic resolution can vary among phytoplankton, invertebrates, and fish due to primer–template mismatches and differential amplification efficiencies, potentially resulting in inconsistent assignments across groups [28]. Uneven completeness and curation of reference databases may further limit taxonomic resolution and contribute to false negatives for certain taxa [29].

This study can also be discussed in the broader context of biodiversity assessments on artificial structures. Recent studies indicate that eDNA metabarcoding can effectively characterize fish assemblages associated with artificial reefs and, when integrated with independent approaches (e.g., acoustic surveys), can provide complementary evidence for reef-associated community patterns and spatial variability [30]. For restored oyster reefs, community composition and functional attributes may differ from those of reference natural reefs during early successional stages, and recovery trajectories can depend on local environmental conditions and restoration age [31]. Such maturation effects are consistent with evidence that multiple reef functions may approach reference levels only after several years of development [32]. Accordingly, repeated eDNA surveys across multiple seasons and years would help distinguish persistent site differences from short-term variability and better resolve maturation trajectories on restored or artificial habitats [29].

### 3.2. Diversity and Community Structure Across Different Marine Ranching Types

Regional differences in phytoplankton community diversity were observed among the three marine ranching areas. In the Tianjin Dashentang Marine Ranching, no significant differences were detected in alpha diversity indices (Richness/Chao1 and Shannon/Pielou) between ranching and control sites, suggesting that artificial construction had limited short-term effects on phytoplankton diversity. In the Furong Island area, species richness was significantly higher at the control site than at the ranching site, whereas Shannon indices were comparable, indicating that the control site was dominated by a few abundant species and exhibited lower community evenness, while the ranching site supported a more balanced species distribution. In the Tianjin Binhai National Marine Park, no significant differences in Alpha diversity were observed between artificial and natural reefs, although natural reefs showed slightly higher evenness, reflecting a relatively more stable community structure. Beta diversity analyses based on PCoA further supported these patterns, with samples from the Dashentang and Furong Island ranching sites clustering more tightly, while those from the corresponding control sites were more dispersed. This suggests that artificial habitats may homogenize phytoplankton community composition by stabilizing local environmental conditions or increasing habitat complexity [33]. Such patterns likely result from multiple interacting mechanisms, as artificial structures and associated organisms (e.g., oysters and macroalgae) can enhance water transparency and oxygen availability and stabilize pH through nutrient uptake and photosynthetic activity [34], while complex substrates may reduce water flow and create localized retention zones that promote microalgal growth and increase water-column diversity [35].

Invertebrate communities exhibited diversity patterns and driving mechanisms distinct from those of phytoplankton across the three surveyed marine ranching areas. In the Tianjin Dashentang Marine Ranching, invertebrate species richness was markedly higher in the ranching zone than in the control area, whereas in the Western Furong Island Marine Ranching Area, alpha diversity indices did not differ significantly between ranching and control zones. In the Tianjin Binhai National Marine Park, differences in invertebrate diversity between artificial and natural reefs were relatively subtle. Spatial patterns of beta diversity further highlighted the consistent influence of artificial structures on invertebrate community composition. PCoA analyses across all three regions showed that sites associated with artificial structures—including ranching zones and artificial reefs—exhibited tighter clustering and greater compositional similarity, while control areas or natural reef zones displayed higher spatial variability. This pattern suggests that artificial reefs may enhance community similarity and stability by providing stable attachment substrates and supplementary food resources [33]. Newly introduced hard substrates increase available habitat for sessile invertebrates such as barnacles, oysters, sponges, and tube worms, while also facilitating the accumulation of organic detritus that supports detritivorous and filter-feeding taxa [16], ultimately leading to more homogeneous invertebrate assemblages within ranching zones.

Alpha diversity of fish communities varied markedly among regions, although marine ranching zones generally exhibited higher species richness and evenness than corresponding control areas. In the Tianjin Dashentang Marine Ranching, no substantial differences were observed between ranching and control zones in Richness and Chao1 indices; however, the number of endemic species in the ranching zone was approximately 1.82 times higher than in the control area, indicating an enhanced contribution to regional biodiversity. In the Tianjin Binhai National Marine Park, The Shannon index in the artificial reef area was higher than that in the natural reef area, despite slightly lower species richness. This suggests that the fish community associated with artificial reefs exhibits relatively higher evenness with fewer species, potentially driven by the stable presence of a limited number of dominant taxa, such as *Planiliza haematocheilus*. In addition, we observed a limited overlap in fish diversity indices between artificial and natural reef areas, which may indicate that the artificial oyster reefs are still at a restoration stage and are influenced by a combination of local environmental conditions and anthropogenic disturbances. In the Western Furong Island Marine Ranching Area, the ranching zone exhibited significantly higher Richness and Chao1 indices than the control area, and several fish species were detected exclusively within the ranching zone. By contrast, higher Shannon and Pielou indices in the control area indicated a more even community structure, whereas the ranching zone supported greater species numbers but relatively lower evenness. Overall, these patterns indicate that marine ranching contributes to increased fish biodiversity, particularly by enhancing species richness and the occurrence of endemic taxa. Furthermore, comparison with historical eDNA data revealed that fish species richness in 2024 exceeded that recorded in 2021, accompanied by a shift in dominant species from *Sardinella zunasi* to *Hypoatherina woodwardi*, reflecting progressive ecological recovery associated with sustained marine ranching development [17].

### 3.3. Environmental Drivers of Community Structure

Environmental gradients play a fundamental role in shaping marine community structure [36]. For phytoplankton, temperature increases can enhance photosynthetic activity but simultaneously intensify nutrient limitation, favoring heat-tolerant dinoflagellates over diatoms and potentially leading to reduced community diversity [37]. Variations in pH further regulate community composition by influencing carbon fixation processes, with alkaline conditions promoting calcifying algae, whereas acidification favors soft-bodied taxa [38]. Changes in salinity and electrical conductivity impose osmotic stress, driving seasonal reorganization of phytoplankton assemblages [39]. As poikilothermic organisms with high physiological sensitivity [40], marine invertebrate communities respond strongly to environmental gradients, particularly temperature, pH, salinity, and electrical conductivity [41]. Elevated temperatures increase metabolic demand but can exacerbate thermal stress [42], while lower temperatures suppress respiratory efficiency [43], collectively contributing to seasonal shifts in distribution. pH variation affects calcification and acid–base balance [44], with low pH conditions inhibiting shell formation in calcifying taxa [45] and higher pH facilitating skeletal growth [46]. Salinity and electrical conductivity further structure invertebrate communities through osmoregulatory constraints [47]. For fish, temperature gradients regulate migration and habitat use [48], while acidification influences gill respiration [49], and variation in salinity and electrical conductivity affects osmoregulatory capacity [50] and neural function [51].

RDA further elucidated the influence of environmental variables, including temperature, pH, salinity, and conductivity, on the community composition of the three major taxonomic groups. For phytoplankton, pH emerged as a key driver in multiple regions. In the Tianjin Dashentang and Binhai Oyster Reef areas, phytoplankton community variation was closely associated with pH, with ranching sites clustering along relatively stable pH gradients in the RDA ordination. This pattern suggests that artificial structures may buffer local pH fluctuations through processes such as nutrient uptake by attached organisms, thereby creating microenvironments conducive to the stable growth of specific algal taxa [52], consistent with observations from the Nan’ao Island Marine Ranch in the South China Sea [53]. In contrast, phytoplankton communities in the Furong Island area exhibited stronger responses to salinity and conductivity gradients, with ranching and control sites separating along the salinity axis, reflecting selective pressures imposed by pronounced salinity variability [54]. For invertebrates, RDA indicated that temperature was the primary factor shaping community variation in the Tianjin Dashentang and Furong Island areas, where ranching sites tended to cluster toward higher temperature values. This pattern implies that artificial reefs may create relatively warmer or more thermally stable microhabitats, favoring the settlement and proliferation of certain filter-feeding invertebrates [55]. In the Binhai Oyster Reef area, however, salinity and conductivity jointly dominated invertebrate community structure, with artificial reef sites shifting along the salinity–conductivity gradient, potentially reflecting changes in local seawater exchange and salt accumulation associated with reef structures [56]. In fish communities, temperature was the primary environmental driver in both the Tianjin Dashentang and Furong Island ranches, with RDA1 explaining 30.25% and 27.33% of the variance, respectively. Fish assemblages in these ranching zones clustered along the temperature vector, indicating a typical thermal niche filtering process [57], consistent with previous eDNA-based monitoring in the Fangchenggang Marine Ranch demonstration area [58]. In contrast, pH was the dominant driver in the Binhai National Marine Park, where RDA1 explained 29.2% of the variance and artificial reef samples exhibited clear displacement along the pH axis, suggesting that artificial reefs may modify local acid–base conditions and thereby exert selective effects on fish assemblages [59].

## 4. Materials and Methods

### 4.1. Study Area and Sample Collection

**Figure 11:**
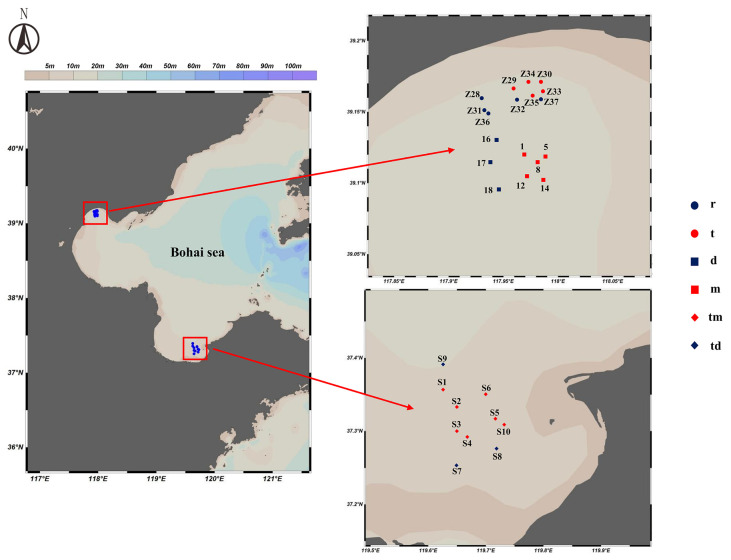
Sampling Station Distribution Map. **r**: Artificial oyster reef area (Tianjin Binhai National Marine Park); **t**: Natural oyster reef area (Tianjin Binhai National Marine Park); **m**: Ranching zone (Tianjin Dashentang Marine Ranching); **d**: Control area (Tianjin Dashentang Marine Ranching); **tm**: Ranching zone (Western Furong Island Marine Ranching Area); **td**: Control area (Western Furong Island Marine Ranching Area).

During May, June, and August 2024, we collected samples at 28 stations across three locations: Tianjin Dashentang Marine Ranching, Tianjin Binhai National Marine Park, and the Western Furong Island Marine Ranching Area (Figure 11). These locations are situated in the Bohai Sea, where hydrodynamic conditions are influenced by the Bohai Sea circulation system. At each station, we collected 2 L of surface water using a 3 L water sampler, producing 28 water samples. We immediately preserved the samples in 2 L amber bottles that had been pretreated with DNA Preservation Solution (Tianenze, Beijing, China) at a 1:10 ratio. The bottles were kept protected from light to prevent DNA degradation from direct sunlight and high temperatures. To minimize contamination during sampling, we rinsed the water sampler with seawater taken from the port side of the vessel, and after thorough rinsing, we collected samples from the starboard side.

At each sampling site, we measured key parameters—temperature, pH, salinity, and conductivity—using a handheld multi-parameter water quality analyzer (YSI 650 MDS, Yellow Springs Instruments, Yellow Springs, OH, USA). We determined water depth with the vessel’s onboard sonar and recorded sampling locations with the ship’s GPS navigation equipment. After collection, we transported water samples to the laboratory for DNA enrichment using Sterivex-GP pressure filter units (pore size: 0.22 μm; EMD Millipore Corp, Billerica, MA, USA). To maximize DNA yield, each sample underwent dual-pass filtration. We expelled residual water from the extraction column with a hypodermic needle and preserved the filter units in DNA preservation solution (Tianenze, Beijing, China). For the negative control, we filtered an equal volume of deionized water to constrain experimental error and confirm the absence of contaminants. After filtration and addition of the preservation solution, we immediately stored samples at −20 °C until DNA extraction. To reduce contamination risk during processing, we disinfected all workbenches and equipment with a 50% bleach solution and re-sterilized workbenches and filtration apparatus with bleach immediately prior to filtration.

### 4.2. eDNA Extraction, PCR Amplification and Sequencing

DNA was extracted with the DNeasy Blood & Tissue Kit (Qiagen, Hilden, Germany) according to the manufacturer’s protocol. Extracted DNA was quantified with a NanoDrop 2000 spectrophotometer (Thermo Fisher Scientific, Inc., DE, Waltham, MA, USA), and extraction blanks were included to detect potential contamination during extraction. Universal primers were used for subsequent amplifications: MiFish-U-F 5′-GTCGGTAAAACTCGTGCCAGC-3′ and MiFish-U-R 5′-CATAGTGGGGTATCTAATCCCAGTTTG-3′ [60] with primer pairs F-5′-ACTGGGATTAGATACCCC-3′ and R-5′-TAGAACAGGCTCCTCTAG-3′ [61] amplified the mitochondrial 12S rRNA gene (12S) to monitor fish in environmental samples. Primers 1380F: 5′-CCCTGCCHTTTGTACACAC-3′ and 1510R: 5′-CCTTCYGCAGGTTCACCTAC-3′ amplified the V9 region (18SV9) of the 18S rRNA gene [62] with primer TAReuk454FWD1: 5′-CCAGCASCYGCGGTAATTCC-3′ and TAReukREV3: 5′-ACTTTCGTTCTTGATYRA-3′ amplification of the V4 region of the 18S rRNA gene (18SV4) [63] was used to monitor phytoplankton and invertebrates in environmental samples [64]. The target gene underwent multiple PCR amplifications following a standard protocol commonly used in eDNA metabarcoding studies. Paired-end sequencing (600 cycles) was performed on an Illumina MiSeq using the MiSeq Reagent Kit V3 according to the manufacturer’s instructions (Illumina, San Diego, CA, USA).

### 4.3. Sequence Quality Control and Clustering

The raw paired-end sequences obtained from sequencing are stored in FASTQ format [65]. For the 12S and 12SV5 raw sequences, primers were first trimmed using cutadapt (v2.3), and then the reads were processed with Vsearch (v2.13.4) [66]. Paired-end reads were merged, underwent quality control, were deduplicated, and had chimeric sequences removed. Operational Taxonomic Units (OTUs) were then clustered at a 97% similarity threshold. To minimize false positives, OTUs with an abundance of 1 and their representative sequences were filtered out. For the 18SV4 and 18SV9 raw sequences, we used QIIME2 (2019.4) [67] with the dada2 workflow to remove primers, perform quality control and denoising, merge paired-end reads, and remove chimeras. Finally, Amplicon Sequence Variants (ASVs) were clustered, and singleton ASVs were filtered out.

### 4.4. Species Identification

For fish communities, species identification was based on OTUs clustered at a 97% sequence similarity threshold from 12S rRNA sequences, whereas phytoplankton and invertebrate communities were resolved at the ASV level using DADA2. After obtaining high-quality OTU representative sequences and ASVs, taxonomic annotation of the amplicon representative sequences was performed using the naive Bayesian classifier (classify-sklearn) in QIIME2 (version 2019.4) [68]. For the phytoplankton community, amplicon sequence variants (ASVs) clustered based on the V4 hypervariable region of the 18S rRNA gene (18S V4) were taxonomically annotated using the nucleotide sequence database (nt) downloaded from NCBI. For the invertebrate community, ASVs clustered based on the V9 hypervariable region of the 18S rRNA gene were annotated using the NCBI nt database. For the fish community, operational taxonomic units (OTUs) clustered based on 12S rRNA sequences were taxonomically annotated using a fish mitochondrial database downloaded from MitoFish [69]. To reduce false positive error, OTUs with single abundance and their representative sequences and ASVs with single readings were excluded from this study. In addition, species that did not belong to the three main categories of phytoplankton, invertebrate and fish were also excluded to avoid potential false positives [70].

### 4.5. Diversity Analysis

Alpha and beta diversity analyses were performed in R (v4.3.3). The vegan package was used to calculate four alpha diversity indices, including Richness, Chao1, Shannon, and Pielou. Richness reflects the number of observed species, Chao1 estimates potential unobserved species based on rare taxa and abundance distribution, Shannon accounts for both species richness and evenness, and Pielou measures the evenness of species distribution. Boxplots were generated using the ggplot2 and ggpubr packages. The Kruskal–Wallis test was used to assess differences in alpha diversity indices between the ranching and control areas. Community dissimilarity between samples was calculated based on the Bray–Curtis distance [71], and PCoA was subsequently performed. In the PCoA plots, 95% confidence ellipses were overlaid to illustrate within-group variation. Permutational multivariate analysis of variance (PERMANOVA) was used to test the significance of differences in community composition between the ranching and control areas [72]. Using the measured environmental data, a community–environment association model was constructed with the vegan package, in which physicochemical parameters (temperature, salinity, pH, and conductivity) were treated as explanatory variables and ASV abundance as the response variable. Monte Carlo permutation tests were applied to assess the significance of each environmental factor, and the explanatory power of the first two RDA axes (RDA1 and RDA2) was reported [36]. Venn diagrams were used to compare the numbers of shared and unique species among different regions [73]. Finally, the long-term effects of marine ranching construction were evaluated by comparing fish eDNA monitoring data from the Furong Island area obtained in July 2021 using the same methodology with the present dataset [74].

## 5. Conclusions

This study applied multi-marker eDNA metabarcoding to systematically assess the diversity and community structure of phytoplankton, invertebrates, and fish across multiple marine ranching areas. By integrating data from regions with distinct ecological characteristics and substantial spatial separation, this approach enabled the identification of consistent biodiversity patterns and key environmental drivers at a broader spatial scale. Overall, species richness and community stability were generally higher in marine ranching areas and artificial reef zones than in corresponding control areas and natural reefs, highlighting the positive ecological effects of artificial structures on habitat enhancement and resource recovery. Phytoplankton communities were primarily structured by pH and salinity, with Chlorophyta and Bacillariophyta dominating across regions, whereas temperature was the dominant factor shaping invertebrate communities, particularly influencing the abundance of Cnidaria and Ciliophora in ranching zones. Fish community structure responded strongly to temperature and pH, with ranching areas supporting a higher number of unique species and greater community evenness; moreover, comparisons between 2021 and 2024 revealed increased species richness and community stability over time. Compared with traditional morphology-based surveys, eDNA metabarcoding substantially improved taxonomic coverage and resolution, particularly for small, rare, or cryptic taxa. By synthesizing data across multiple marine ranches, this study not only characterizes site-specific biodiversity features but also demonstrates the utility of eDNA-based approaches for standardized, large-scale biodiversity monitoring in marine ranching systems.

## Data Availability

The original contributions presented in this study are included in the article and the Supplementary Materials. Further inquiries can be directed to the corresponding authors.

## Supplementary Materials

The following supporting information can be downloaded at: https://www.mdpi.com/article/10.3390/ijms27031483/s1.
